# Scraps

**Published:** 1887-03

**Authors:** 


### SCRAPS

PICKED UP BY THE ASSISTANT-EDITOR.

---

**A Green Old Age.**—A town councillor in an ancient borough, in a speech at a meeting called in support of a public movement, said: "I think I may be excused from taking a very active part in this question, as I am an octo-geranium."

**Prophylaxis.**—"So Doctor Pellett is dead?" "Yes. He was an ornament to his profession. He has saved a great many lives." "Why, I didn't know that he had any practice to speak of." "He hadn't; he saved life in another way." "How was that?" "By dying so young."

**A very Broad Hint.**—A physician in this city is said to have the following inscription on his bill-heads: "A patient's gratitude to his doctor is part of his disease, and is most declared when the fever is highest, cools off during convalescence, and entirely disappears with the complete return of health. All bills due upon presentation. Office prescriptions and attendance strictly cash."—*The Medical Record* (New York).

**An Opportunity for Bath.**—It is somewhat strange that Bath has not followed the example of other towns by devoting one Sunday in the year to the benefit of her hospitals. The Rev. M. E. Hoets, in advocating, the other day, the claims of the old Mineral Water Hospital, alluded to this want—a want which, it is to be hoped, will soon be supplied. Will not the good citizens of Bath come forward and help to establish a Hospital Sunday Fund? Surely philanthropy in Bath ought not to lag behind that of smaller and more humble towns.—*The Hospital.* [Perhaps, since this was written, a day has been set apart for the purpose.]

**Novelists' Medicine.**—Lady writers of fiction, as a rule, limit their literary eccentricities to excursions amongst the amorphous elements of "Novelists' French" and un-English grammar. They sometimes deal freely with poison and the dagger, but rarely venture upon strictly anatomical details. The most unfortunate *lapsus calami*, however, which has come under our observation was thus incurred. The hero, with great difficulty, has succeeded in saving the heroine from falling down the precipitous side of a mountain on which they have lost their way. The lady has fainted, and is apparently lifeless. But, to his intense relief, the gentleman discovers, "by the pulse in her femoral artery," that the heart still beats.—*Medical Times.*

**A Pharmaceutical Term of Reproach.**—Our readers are all doubtless informed as to the brilliant war of words that once took place between a Billingsgate fishwoman and a scholar, in which the latter made use of mathematical terms that his adversary could not match; not precisely in the same line, but evidently quite as effective, was an expression of disgust lately applied by a woman to a Paris policeman: "Tu me fais l'effét d'une pilule." This pharmaceutical abuse was more than the policeman could endure, and the woman was brought before one of the police courts, where, according to a Paris despatch to the London *Daily Telegraph*, she was acquitted, on the ground that there were a thousand kinds of pills, the effects of which were of the most varied character, but she had not mentioned any particular kind. "So we may infer," the account continues, "that, had Ernestine Roussel compared her enemy to a blue pill, for instance, she would have been treated with more rigor."—*New York Medical Journal.*

**Caius.**—Dr. Caius (1510–1573) showed himself notably before his age in his censures of excess in eating and drinking, his commendation of the bath and of muscular exercise. His advice to his readers to have recourse to a good physician, and to be at least as good to their bodies as to their hose or their shoes, is followed by a picture of the army of quacks who in default of science preyed upon the masses. "Simple-women, carpenters, pewterers, braziers, soap-ball sellers, apothecaries, avaunters themselves to come from Pole, Constantinople, Italy, Almaine, Spain, France, Greece, Turkey, India, Egypt, or Jury; from the service of emperors, kings and queens, promising help of all diseases, yea incurable, with one or two drinks, by drinks of great and high prices, as though they were made of the sun, moon, or stars; by blessings and blowings, hypocritical prayings, and foolish smokings of shirts, smocks, and kerchiefs, with such others, their phantasies and mockeries, meaning nothing else but to abuse your light belief, and scorn you behind your backs, with their medicines (so filthy that I am ashamed to name them), for your single wit and simple belief, in trusting them most, which you know not at all and understand least; like to them which think far fowls have fair feathers, although they be never so evil favoured and foul; as though there could not be so cunning an Englishman as a foolish running stranger, or so perfect health by honest learning as by deceitful ignorance."—BETTANY'S *Eminent Doctors.*

**The Early Formation of Character.**—T. C. M., in *The Cincinnati Lancet and Clinic,* thus translates from the French:

"Within the deep interior of a womb,
A narrow and contracted dwelling-place,
Two embryos had found a living tomb,
Although their getting there was no disgrace.
The first of these head downward hung,
And to his brother's legs strong hugs would give;
While every day a merry song he sung—
'How happy 'tis a fœtal life to live!

"'Here in our jolly home is never care,
Plenty around us casts a generous store;
Oh, let us always be a merry pair;
How charming thus to live for evermore.
Come! swim upon the amniotic tide,
And each placental bumping e'en forgive:
What joy it is upon one's back to slide;
Oh, happy 'tis a fœtal life to live!'

"The second twin, head pointed to the sky,
Cast looks disdainful on the one below,
And answered with a mournful fœtal sigh—
'I like you; but you are a little slow.
You laugh, and frisk about like a weak child
Who in the simplest joy doth pleasure find:
By such a living I am not beguiled;
My life's unhappy, save among mankind.

"'Here in this uterine dungeon, dark, confined,
This gloomy prison cell so moist and close,
I knock my head until I'm nearly blind,
While your big feet are kicking 'gainst my nose.
If I perchance some gentle movement make,
You use my navel string for jumping rope.
Your company I'd really like to shake;
I long for death, it seems my only hope.'

"E'en as he spoke the watery sac gave way,
The womb was wrapped in labor's painful throes;
The under twin slipped into light of day,
A-squalling sadly, 'Now commence my woes.'
As for the one who hated uterine life,
He bawled a song of praise unto the Lord,
And smiled serenely as the old midwife
Put on a belly-band o'er his clipped cord."

